# Assessing Aquatic Readiness as a Health-Enhancing Measure in Young Swimmers with Physical Disabilities: The Revised Aquatic Independence Measure (AIM-2)

**DOI:** 10.3390/ijerph22030421

**Published:** 2025-03-13

**Authors:** Anat Chacham-Guber, Yadin Sapir, Aviva Goral, Yeshayahu Hutzler

**Affiliations:** 1 Israel ParaSport Center, Ramat-Gan 5253529, Israel; anatc@iscd.com (A.C.-G.); yadin@iscd.com (Y.S.); 2 The Exercise Physiology Program, School of Public Health, Faculty for Medical and Health Sciences, Tel Aviv University, Tel Aviv 6997801, Israel; 3 Levinsky-Wingate Academic College, Faculty for Movement and Sports Sciences, Wingate Institute, Netanya 4290200, Israel; avivag@l-w.ac.il

**Keywords:** motor function, physical disability, learn to swim, motor tests, training

## Abstract

Based on the well-established health outcomes associated with the aquatic environment, this study aimed to evaluate the validity of the Aquatic Independence Measure—Revised (AIM-2). The original scale comprised twenty-three skills, graded on a five-point proficiency scale. A sample of one hundred eight young swimmers with disabilities, who had completed at least two years of training, was assessed by their coaches, generating a dataset of four thousand nine hundred sixty-eight scores. Exploratory factor analysis (EFA) and internal consistency measures were applied to validate the scale’s structure, resulting in the extraction of three factors accounting for sixty-four percent of the variance, based on thirteen original items. Two independent experts established interrater reliability in twenty-two participants. Additionally, divergent validity was examined across participants’ years of swim-training experience across three disability severity categories, gender, and two age groups. Significant differences were found in skill acquisition based on years of experience and disability severity. The results indicate that the AIM-2 effectively assesses swimming readiness in young swimmers with disabilities. Coaches can use it to monitor progress, optimize training, and support the health benefits of aquatic activities for children and adolescents with disabilities.

## 1. Introduction

Swimming and aquatic interventions have long been valued for their physiological benefits among individuals with neuromotor impairments—both adults [1,2] and children [3,4]. The unique advantages of aquatic environments have been highlighted, following their favorable outcomes compared to those of land-based exercise. These include (a) water viscosity and hydrostatic pressure, which reduce the straining effect of gravity on impaired soft tissues [1,3]; (b) the hydrostatic pressure, which enhances oxygen transportation throughout the cardiorespiratory system—in turn, improving the outcomes of aquatic exercise [1,5,6,7]; (c) buoyancy, which enables the initiation of independent movements that are challenging to perform on land, mainly because of gravitational constraints [7,8]; and (d) turbulence, which reduces muscle tone—thereby promoting physical efficacy in individuals with neuromotor impairments [1].

In children with disabilities, programs that incorporated aquatic exercises were found to yield superior results compared to those that relied solely on land-based physical activities [7,9,10]. In their systematic review, Roostaei and associates [11] found that aquatic exercise in children with cerebral palsy is feasible, with no adverse effects being seen; yet dosing parameters remain unclear. Aquatic exercise enhances functional performance on land while providing an enjoyable, adaptive, and accessible activity that supports mental and social wellbeing [12,13]. Indeed, learn-to-swim programs were found to be among the main aquatic interventions for children with diseases of the nervous system [14]. These aquatic interventions are typically designed to promote autonomous functioning (i.e., aquatic independence) in the water and acquiring swimming patterns. Given the potential of such interventions to enhance the participants’ quality of life, assessing aquatic independence and swimming competence offers a valuable tool for developing and maintaining learn-to-swim programs for children with physical disabilities.

A range of studies have explored tools for assessing swimming skills. The Canadian Red Cross [15,16] and YMCA [17], for example, have published widely recognized guides on learn-to-swim progress. These guides focus on water orientation—such as entering and exiting the water, breath control, and buoyancy—as well as on gliding, arm and leg actions, and the coordinated use of limbs for performing specific swimming strokes. Thomas [18] provides a comprehensive guide for teaching swimming strokes and skills, with a scoring system for gauging success. Killian and associates [19,20] developed and validated a checklist for assessing water orientation and beginner swimming skills in children with autism. Gelinas and Reid [21] argue that swimming skill development in typically developing children may not apply to children and adolescents with physical disabilities (CAWPDs), who may progress at a different rate and sequence.

In 2000, these researchers developed an adapted test for individuals with developmental disabilities, especially those with limitations to their movement system. However, they have used a fail/pass system, whereas a tiered (e.g., five-point) grading system is argued to provide more objective information [22] and to motivate students to perform better than they would with a simple pass/fail system [23].

Research highlights the importance of formal instruction in learn-to-swim programs for children and adolescents. Formal training enhances skill development and helps to prevent drowning [24]; it is also associated with higher self-reported swimming proficiency compared to that by informal learning [25]. Swimming programs were reported to be effective in improving water safety and skills in children and adolescents with disabilities, including those with autism-spectrum disorders and severe motor impairments [26,27,28]. Yet assessment tools are crucial for aquatic skill programs, as these ensure effective skill acquisition and development while preventing overconfidence that could lead to dangerous situations [29]. Reliable assessments also help to quantify basic aquatic skills while estimating the children’s developmental stage [30]. These tools should include fundamental aquatic movement skills, which are often overlooked in traditional taxonomies of such abilities [31]. In their systematic review, Santos-Garcia and colleagues identified a shortage of validated measurement instruments for measuring the overall aquatic competence in infants and elementary school children [32]. Two newer scales have also been published and validated [33,34], yet their items and scoring seem to lack suitability for evaluating CAWPDs, where a dedicated aquatic competence scale is greatly needed [27,35].

In 1998, Hutzler et al. [7] developed the Water Orientation Scale (WOS) for children and adolescents with disabilities. Later renamed the Aquatic Independence Scale (AIM), it was further validated in an additional study [36,37]. The revised AIM-2 version, introduced about a decade ago, has since been examined in various contexts. Since its emergence, the tool has been widely used by the Israel Sport Center for the Disabled (recently renamed the Israel Parasport Center) for young individuals with a variety of physical disabilities, including poliomyelitis and cerebral palsy [38].

Test validity, i.e., “the degree to which evidence and theory support the interpretations of test scores for proposed uses of the test” [39], p. 11, is possibly the most fundamental consideration when developing an evaluation tool. Construct validity verifies the relationship between a given measure and a range of specific variables—whether the association is positive, negative, or lacking [40]. This study aimed to examine the construct validity of the AIM-2 scale, which assesses swimming skills in beginners with physical disabilities and no prior swimming experience. The primary goal was to investigate the structure of the test and its internal reliability, while the secondary goal was to examine its divergent validity. We assumed that the instructors’ ratings of the participants’ swimming skills would differ by disability level, age, and gender. Furthermore, we assumed that all the categories should improve with time. For this study, the following research hypotheses were defined:

1. Following a year of training, the overall level of performance in swimming functions—in general and for each generated factor—would be higher. To the best of our knowledge, this is the first study to compare aquatic performance across different levels of disability;
2. The more severe the level of the disability, the lower the performance of the swimming functions;
3. Based on the more frequent participation of male children and adolescents in sports, it was expected that they would score higher than females [41];
4. Older participants would perform better and acquire swimming skills faster than younger participants.

To the best of our knowledge, this is the first study to compare aquatic performance across different levels of disability.

## 2. Materials and Methods

To conduct this validation study, a retrospective database of rating scores was used, using the AIM-2 tool and collected over six years.

### 2.1. Item Pool Construction

The AIM-2 was developed based on items that appear, in part, in previously existing tests, such as those of the Canadian Red Cross [15,16], YMCA [17], and Killian et al. [19,20]. A panel of five experts in aquatic therapy, with 7–25 years of experience, assessed the relevance of each item that was included in the new scale; this resulted in a twenty-two-item assessment tool [36,37]. Over the years, some items were removed, while others were added. During the six years explored in this study, the scale comprised twenty-three items. Each item was rated based on the degree of assistance the child requires for performing a given task, on a scale from 0 (does not initiate the task) to 4 (completes the task independently without assistive flotation devices). The total scale score was calculated as the mean sum of all the remaining items following the exploratory factor analysis (described below). Similarly, the score of each factor was calculated as the mean of all the items that were loaded onto that factor.

### 2.2. Procedure

#### 2.2.1. Recruitment and Data Collection

The data gathered in this retrospective study are based on assessments that were conducted by swimming instructors during 2015–2020 at a major sports center for persons with disabilities. Each year, the swimming assessments were conducted over a four-week period during the same month. The inclusion criteria consisted of participants who had (1) been appraised for at least another year following the first assessment, (2) were aged 4–23 years, and (3) had participated in at least 50% of the training sessions during the month leading up to each assessment. The final sample size included 108 participants. All the raters were experienced hydrotherapists and aquatic instructors, who were trained to rate the participants’ performance in each item under the supervision of the scale developer.

In addition to the 23 items from the AIM-2 tool, gender, age, and disability level were also documented. For statistical purposes, two age groups were defined: children, aged up to 11 years (i.e., the younger group) and adolescents, aged 12 years and older (i.e., the older group). Swimming requires the coordination of leg, trunk, arm, and head movements against water drag and was found to be associated with gross motor function, such as walking, in children with cerebral palsy [37,42]. The disability level was, therefore, determined based on the participants’ walking capability, which was coded as (1) mild, able to walk independently and manage their own self-care; (2) moderate, uses walking aids, such as a walker or crutches, and needs some self-care support; and (3) severe, requires a wheelchair and/or full support for managing their self-care. The assessment followed a strict procedure, including instructions on what to do, the instructor’s demonstration of the task, and a preliminary trial, if it appeared to the instructor that the child did not understand the task. This study was approved by the ethics committee at the researchers’ affiliated academic institution (Levinsky-Wingate Academic College).

#### 2.2.2. Analytical Procedures

##### Exploratory Factor Analysis (EFA)

To achieve a working version of the scale, EFA was performed on the 23 items. Principal axis factoring with a varimax rotation was employed, consistent with standard scale construction procedures [43,44]. EFA was conducted in several iterations, each leading to the removal of one or more items with low loading values (<0.4), multiple loadings (>0.4) in two or more factors, or items that formed single-item factors. This process was repeated until no more items were removed. The final number of factors was determined based on the factors’ eigenvalues (>1.0) and supported by the scree plot. Sample adequacy for EFA and the chosen rotation technique was evaluated using the Kaiser–Meyer–Olkin measure, the diagonal of the anti-correlation matrix, and the factor transformation matrix. These criteria were all satisfied with the current data. Statistical analyses were performed using SPSS v. 29 (IBM, Inc., Armonk, NY, USA).

##### Validity Analysis

Using the final version of the scale following the EFA, descriptive statistics were conducted for the overall scale and each factor (i.e., internal consistency). An inter-rater reliability check was also conducted, using intra-class correlation analyses between two expert aquatic therapists (one holding a Ph.D.; the other, an M.Sc.) with more than 30 years of accumulated experience. The data included 22 participants—for the overall scale and each factor. Finally, the scale construct’s validity was assessed using statistical analyses, such as *t*-tests and repeated measures ANOVA for comparing the total scale and subscale mean scores by year (first or second), disability level (mild, moderate, or severe), and gender. ${\eta_{p}}^{2}$ was computed to indicate the effect size.

## 3. Results

### 3.1. Participants

The 108 participants who were included in this study were aged 4–23 years at the time of the first assessment (M = 11.07 years; SD = 4.14 years). A total of 57 (52.8%) were boys, 23 (21.3%) were classified as having a moderate disability, and 31 (28.7%) were defined as having a severe disability (Table 1). Significant mean-age differences were seen between disability levels (F_2,105_ = 11.47, *p* < 0.001), with post hoc analysis indicating a significantly higher mean age among participants with a severe disability than with a moderate (*p* = 0.013) or mild (*p* < 0.001) disability. Almost half (46%) of the participants who were aged 12+ years were severely disabled, compared to only 13.8% of those who were aged 4–11 years, suggesting an inverse relationship (Table 1). No significant differences were found between boys and girls by disability level. Learning difficulties were reported in 17 participants (15.7% of the study population).

### 3.2. The AIM-2 Scale and Factor Structure

**Figure 1:**
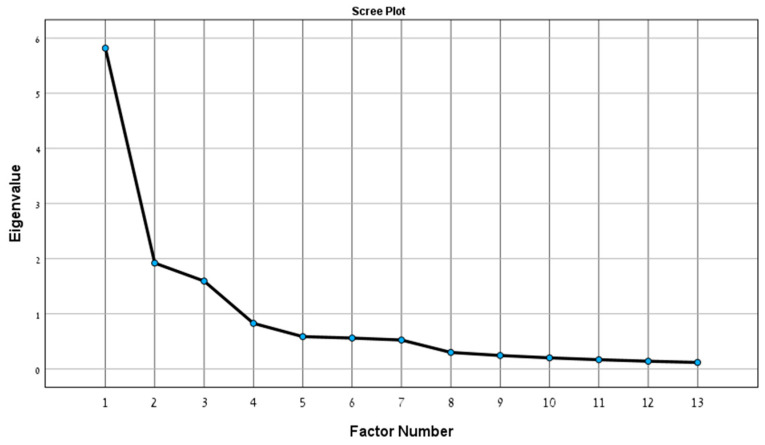
Scree plot for exploratory factor analysis (EFA) of the AIM-2 scale.

The Kaiser–Myer–Olkin value (KMO = 0.803) and Bartlett’s test of sphericity (X^2^ = 934.22, *p* < 0.001) for the 23-item pool model were high, indicating good internal consistency, which was sufficient for the EFA. Of the 23 items, 13 were retained following the EFA. Based on eigenvalues and scree plot analyses, a three-factor structure was observed, with each factor being measured by at least three items—as recommended for scale construction [45]. The key EFA components are presented in Table 2 (eigenvalues) and in Figure 1 (scree plot). These three factors were labeled as (1) adaptation (six items), (2) basic skills (four items), and (3) advanced skills (three items). These are referred to in this article as the AIM-2 subscales. Reliability levels were high for the total scale (13 items; α = 0.890) and for all three factors [46], with the latter accounting for 64% of the explained variance.

### 3.3. Correlations Between the Total AIM-2 and Subscale Scores

Strong significant correlations were seen between the total AIM-2 scale and the three subscales. For the first-year assessments, low to moderate correlations [47] were seen between the three subscales (r values ranged between 0.379 and 0.524; *p* < 0.001 for all the correlations), and even stronger correlations were seen for second-year assessments, i.e., moderate to strong (ranging between 0.532 and 0.857). Further associations are presented in Table 3.

### 3.4. Two-Year Data Comparisons

#### 3.4.1. ANOVA Results by Time and Disability for the AIM-2 Scale and Subscales

Table 4 presents both descriptive and inferential statistics for the AIM-2 mean scores by year and disability level. The results show significant main effects of time and disability level on the total scale and two subscales (basic skills and adaptation). The mean scores improved in the second year across all the disability levels. Moreover, the more severe the disability level, the lower the AIM-2 mean scores, with a significant difference being seen between participants with a severe disability and those with a mild or moderate disability (*p* < 0.001 for both). No significant differences were seen between the mean scores of the participants with a mild or a moderate disability.

A significant main effect of the disability level was found across time for the total scale and all three subscales, with lower scores in participants with severe disabilities and higher scores in those with mild disabilities. Finally, a significant year X disability-level interaction was observed for the adaptation subscale (*p* = 0.01). The increase in AIM-2 mean scores over time was the highest among the participants with a moderate disability level compared to those with a mild or severe disability.

#### 3.4.2. ANOVA Results by Time and Gender for the AIM-2 Scale and Subscales

Mean comparisons by time and gender show a significant main time effect for the total AIM-2 scale and for all three subscales (Table 5), whereby means were significantly higher in the second year than in the first one. In addition, means for the total scale and for the advanced-skill subscale were significantly higher among males than among females. No significant interaction was found.

#### 3.4.3. ANOVA Results by Time and Age for the AIM-2 Scale and Subscales

**Figure 2:**
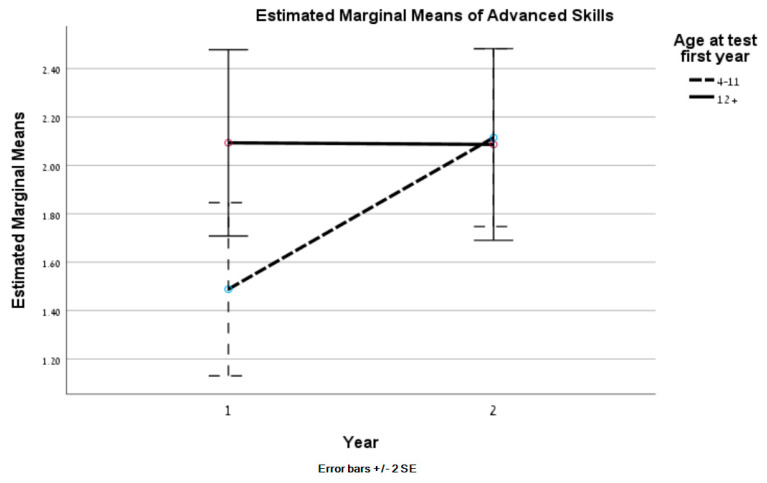
Advanced skills: means by year and age group.

Mean comparisons by time and age showed a significant main effect of time for the total AIM-2 scale and all the subscales, with higher scores in the second year across both age groups (Table 6). Moreover, adaptation means were significantly higher among younger participants (4–11 years) compared to older ones (12+ years). In addition, a significant interaction was found for the advanced-skill subscale. As seen in Figure 2, the mean score for the advanced-skill factor greatly increased in the second year among younger participants; yet this pattern was not seen in the older age group.

#### 3.4.4. Inter-Rater Reliability and Yearly Score Correlations

An inter-rater reliability analysis was performed on a small sub-sample of the participants (n = 22), who composed 20% of the whole sample. In this sub-sample, very strong (0.99) and significant (<0.001) intra-class correlations were observed for the total AIM-2 scale, as well as for all three subscales. In addition, significant correlations were found between the first and second years in the total AIM-2 scale and subscales. A moderate correlation (r = 0.730) was found between the first and second years’ total scores (Table 7). The strongest correlation was found for the adaptation subscale (r = 0.813), while the weakest correlation was seen for the basic-skill subscale (r = 0.476). All the correlations were significant (*p* < 0.001 for all the correlations).

## 4. Discussion

This study aimed to investigate the construct validity and responsiveness of the AIM-2 Scale, designed for conducting annual assessments of aquatic competence in young individuals with a physical disability. Building on these results, the key aspects of the tool will be examined, including its internal structure, construct validity, discriminant capability, and responsiveness to change.

### 4.1. Internal Structure

These findings revealed a valid construct, suggesting a thirteen-item, three factor (subscale) composition (adaptation and basic and advanced skills), which explained sixty-four percent of the variance. The inclusion of thirteen items on an assessment scale is within the range of typical aquatic competence tools for able-bodied children (from six to nineteen items) [32]. Using the original AIM scale, a similar three-factor solution (water rotation and adjustment, floating abilities, and swimming skills) was also seen in a sample of forty-nine young children with cerebral palsy [37]. Moreover, the findings of the current study support the logic behind the scale construction, as stronger associations were seen between basic and advanced skills than between adaptation and basic skills; this suggests that aquatic adaptation may be a more distinct category within the skill hierarchy.

In the smaller sub-sample (n = 22), inter-rater measurements were highly associated across the full AIM-2 scale and all three subscales. This strong consistency suggests that the AIM-2 measures demonstrate reproducible reliability between assessments. Similarly, high-level associations were also seen in the original AIM for young children with cerebral palsy [36] and for able-bodied children who were assessed via a different learn-to-swim assessment tool [48]. In the current study, moderate to strong correlations were seen between the first and second years of assessments, thereby further demonstrating a valid internal structure.

### 4.2. Construct Validity

In this study, the construct validity was established by examining theoretical hypotheses related to time, disability level, and gender, as detailed in the following sections.

#### 4.2.1. Performance Across Time

In mainstream learn-to-swim programs, the number of lessons attended was found to be a stronger predictor of skill acquisition than factors such as gender or age [49]. However, although many aquatic programs for CAWPDs have reported improvements in fitness and motor skills [4], see reviews in: [35], few have specifically documented gains in swimming skills [7,50]. The current study contributes to the understanding of skill acquisition trajectories in relation to adaptation and basic and advanced aquatic skills. We expected an improvement in skill acquisition within a year of training. Indeed, after one year of training (about 10 months), significant and large improvements (${\eta_{p}}^{2}>0.15$) were seen in the participants’ adaptation and basic skills; yet only modest progress was seen in advanced skills, possibly not achieving the goal of aquatic independence. These results suggest that the journey to achieving swimming independence in CAWPDs may be a prolonged process, extending beyond a single year or even two subsequent years. It has been suggested that advanced swimmers spend more time in the propulsive phase and show greater consistency in relative timing [51]. For swimmers with a disability, and particularly those with a severe disability, it would probably take a longer time to develop the strength needed to increase the propulsive phase. Strategies to support swimmers with a disability to develop propulsive strength may include on-land rubber band or resistive pulley training, as well as water training, such as with gloves and tie ropes [52].

#### 4.2.2. Performance Across Disability Levels

The literature demonstrates promising outcomes in motor-skill development through targeted interventions for children and adolescents with physical disabilities [53]. However, in CAWPDs, such as those with cerebral palsy, both qualitative and quantitative impairments in skill acquisition have been observed. These deficits are especially pronounced in implicit motor-sequence learning compared to their typically developing peers [54]. Donaldson et al. [55], for example, found that children with a developmental coordination disorder significantly underperformed compared to their age-matched peers in all water competency tasks and in the front crawl. Nevertheless, both groups demonstrated significant improvement in water competency and the front crawl following ten lessons—indicating that structured swimming instruction can be beneficial for all participants, regardless of their initial skill levels.

We hypothesized that performance would be correlated with the severity of the disability. Our findings demonstrate that although there was overall improvement over time across all three disability levels—except for advanced skills—a significant and gradual effect was seen between the three disability categories, with large effect sizes for adaptation and the total scale. However, the lack of an interaction effect between time and disability suggests that despite variations in performance scores, the learning trajectories followed similar patterns across all the groups. Overall, our findings reveal a strong relationship between the severity of the disability and individual performance trajectories in aquatic skills. Participants with severe disabilities, who require high levels of support, have been reported to take longer to enter and exit the pool compared to non-disabled swimmers [56]. This extended transition time may reduce the actual training duration in each session, potentially limiting the development of advanced skills that require prolonged practice and exposure.

#### 4.2.3. Performance Across Genders

Gender differences in psychomotor skills related to swimming are evident among children, with girls demonstrating more advanced abilities in body balance, water balance, and overall coordination [57]. In a study on sports education students, however, male participants were found to achieve higher scores in swimming-style learning outcomes, including breaststroke, butterfly, and freestyle [58]. These findings indicate that gender differences exist in swimming acquisition and performance, potentially influenced by age and other factors. Notably, the current study appears to be the first to analyze swimming skill performance across genders in CAWPDs. The findings show a nuanced result, partially supporting our hypothesis: Males performed better only in advanced skills and the total AIM-2 scale but not in adaptation or basic skills. In addition, no interaction was found between time and gender, indicating that there were no differences in the learning effect. It could, therefore, be suggested that the difference observed in performance may be attributed to physical strength [59], more exposure to physical activity, or other background variables attributed to males.

#### 4.2.4. Performance Across Age

The impact of age on swimming skill acquisition has been documented in young children, aged 3–6 years, with older children typically demonstrating better performance. However, as children mature, the influence of age decreases, as experience begins to play a more critical role in aquatic competence [60]. In the current study, a wide age range was assessed to enable evaluation among different age groups. We hypothesized that older participants would perform better than young ones. The results indicate a limited impact of age, with significant differences only being seen in adaptation skills and with a time × age interaction in advanced skills. Notably, although the younger age group showed progress from the first year to the second one, on average, the older age group did not. Research on swimming skill acquisition in non-disabled children reveals mixed findings regarding age and speed. Based on swimming instructors’ testimony, it has been suggested that children aged 8–13 years (referring to most of our younger age group) may learn faster than older ones [61]. Nevertheless, individual training planning is necessary to cope with the mixed impact of age, gender, and disability

#### 4.2.5. Limitations and Future Research Directions

Exploratory factor analysis (EFA) is a fundamental tool for examining the structures of assessment tools and other theoretical constructs [62]. However, it has limitations, particularly regarding sample size and the interpretation of factor solutions. In this study, we ensured a sufficiently large sample and exercised caution in interpreting the factor structure [62]. Nonetheless, despite the relatively large sample compared to previous assessments of swimming in CAWPDs, certain factors may have introduced confounding effects. The broad age range, varying severity of disabilities, and a 15.7% comorbidity rate of learning difficulties among the participants may have influenced the findings. Additionally, the voluntary nature of the participation could have led to selection bias [63], with individuals who have had higher motivation being more likely to enroll.

Variability in instructor assessments may also have contributed to inconsistencies in evaluation. Although all the evaluations were conducted and reported in real time, the retrospective design spanning multiple years presents a potential limitation. However, Powell and Sweeting [64] suggest that although retrospective studies rely on previously collected data, patient case notes from past interventions are generally reliable. Our study utilized real-time assessments conducted in the swimming pool, following a strict and comprehensive procedure to minimize inconsistencies. Despite the strong inter-rater agreement, minor variations in evaluations may have occurred, particularly among less experienced trainers or those susceptible to expectancy bias. Such bias can result in assessments being influenced by prior expectations or general impressions of participants [65,66]. To mitigate these concerns, future research should incorporate extended training periods to ensure consistency in skill evaluation.

Further validation of the revised scale should be pursued in future studies by incorporating additional variables. For example, assessing the correlation between swimming scores and the Gross Motor Function Measure in children with CP, as examined in the original version [37], would be valuable. Moreover, evaluating the test items in children and adolescents with other disabilities, such as intellectual disabilities and autism, would enhance the scale’s applicability. Investigating its utility and validity in adults with acquired disabilities, who are relearning to swim, represents another important research direction.

Finally, future studies should explore the impacts of innovative skill-learning programs, including strength-training interventions [52] and non-linear pedagogical approaches [67], to further enhance skill acquisition and performance outcomes.

## 5. Conclusions

This study explored the structure and construct validity of the AIM-2 scale for assessing swimming skill acquisition in CAWPDs; a focus was placed on the assessment time (first and second years), disability level (mild, moderate, or severe), age (child or adolescent), and gender as key factors. The findings of this study indicate that the Revised Aquatic Independence Scale is a valid and reliable tool that can be used to assess the swimming skills and development of CAWPDs. Furthermore, the findings indicate that after one year of training, participants showed significant improvements in adaptation and basic swimming skills, while progress in advanced skills was more limited. Achieving swimming independence for CAWPDs may require more than two years of training, with performance being strongly linked to the severity of the disability. This aligns with previous studies whereby motor skill development is slower in children with disabilities.

In terms of disability impacts, this study found that participants with more severe disabilities demonstrated lower overall performance. However, the absence of interaction effects between time and disability suggests that although performance levels differ, the learning trajectories across disability groups are relatively similar. Gender analysis revealed that boys outperformed girls in advanced skills, but no significant differences were observed in adaptation or basic skills. This may be because of strength differences, as suggested in the literature.

## Figures and Tables

**Table 1:** Descriptive statistics by disability level.

Parameter	Disability level			Total
	Mild	Moderate	Severe	
Gender (n, %)				
Boys	14 (13.0%)	13 (12.0%)	30 (27.8%)	57 52.8%)
Girls	24 (22.2%)	10 (9.3%)	17 (15.7%)	51 (47.2%)
All	54 (50.0%)	23 (21.3%)	31 (28.7%)	108 (100%)
Age (mean, SD)	9.59 (3.31)	11.04 (3.91)	13.68 (4.42)	11.07 (4.14)
Age distribution				
4–11	36 (62.1%)	14 (24.1%)	8 (13.8%)	58 (100%)
12+	18 (36%)	9 (18%)	23 (46%)	50 (100%)

**Table 2:** EFA results for 23 AIM-2 items.

No.	Item	Factor Loading		
		1 Adaptation	2 Basic Skills	3 Advanced Skills
4	Walking across the pool at waist height	0.821		
1	Entering the water	0.773		
3	Walking along the wall	0.733		
2	Holding the pool’s rail	0.659		
15	Changing flotation position prone/supine	0.651		
5	Exhaling five times in the water	0.580		
21	Swimming a 15 m backstroke		0.846	
13	Progressing 4–5 m in deep water		0.761	
23	Exiting the pool		0.713	
19	Swimming a 15 m front crawl		0.698	
22	Swimming a 25 m front crawl			0.846
17	Swimming a 15 m breast stroke			0.783
18	Completing an 8 m crawl arm stroke			0.619
Eigenvalues		5.82	1.92	1.59
Variance percentage (%)		25.95	21.33	16.71
Cronbach’s alpha		0.876	0.879	0.823
Factor mean (SD)		3.85 (1.19)	1.52 (1.56)	1.89 (1.50)
Range		0.33–5.00	0.00–4.50	0.00–5.00

**Table 3:** Correlations between the total AIM-2 and subscales (*n* = 108).

		First Year				Second Year			
Measure		1	2	3	4	1	2	3	4
1	Total AIM-2 scale	--				--			
2	Adaptation	0.853	--			0.857	--		
3	Basic skills	0.831	0.524	--		0.850	0.532	--	
4	Advanced skills	0.671	0.379	0.412	--	0.790	0.553	0.560	--

All the correlations are significant at *p* < 0.001.

**Table 4:** Repeated measures ANOVA results by year and disability level.

Scale	Mild (n = 54)		Moderate (n = 23)		Severe (n = 31)		$F_{\left(1,106\right)}\;\;p\;({\eta_{p}}^{2})$		
	M	SD	M	SD	M	SD	Time	Disability Level	Time X Disability-Level Interaction
**Total scale**							38.94 <0.001 (0.271)	21.18 <0.001 (0.287)	NS
First year	3.11	0.95	2.64	0.94	1.87	0.97			
Second year	3.61	0.88	3.29	0.72	2.28	1.28			
**Basic skills**							30.27 <0.001 (0.224)	5.92 0.004 (0.101)	NS
First year	1.87	1.70	1.54	1.41	0.90	1.21			
Second year	2.83	1.70	2.47	1.43	1.78	1.40			
**Advanced skills**							NS	3.27 0.04 (0.059)	NS
First year	1.88	1.45	1.88	1.46	1.48	1.22			
Second year	2.45	1.47	2.10	1.19	1.49	1.22			
**Adaptation**							29.37 <0.001 (0.219)	46.24 <0.001 (0.468)	F_(2,105)_ = 4.46 0.01 (0.078)
First year	4.55	0.65	3.75	1.03	2.71	1.18			
Second year	4.72	0.37	4.44	0.60	3.01	1.47			

Note: F is the statistic representing the ratio of the mean square between groups to the mean square within groups (with 1 and 106 degrees of freedom unless stated otherwise); *p* is the *p*-value, and (${\eta_{p}}^{2})$ is the partial-eta-squared value, which indicates the effect size. NS = non-significant at a 95% confidence level.

**Table 5:** Repeated measures ANOVA results by year and gender.

Scale	Male (n = 57)		Female (n = 51)		$F_{\left(1,106\right)}\;\;p\;({\eta_{p}}^{2})$		
	M	SD	M	SD	Time	Gender	Time X Gender Interaction
**Total scale**					41.45 <0.001 (0.281)	4.32 0.04 (0.04)	NS
First year	2.78	1.08	2.51	1.08			
Second year	3.42	1.07	2.88	1.13			
**Basic skills**					34.68 <0.001 (0.247)	NS	NS
First year	1.67	1.58	1.36	1.54			
Second year	2.77	1.58	2.10	1.59			
**Advanced skills**					5.91 0.02 (0.053)	3.90 0.051 (0.036)	NS
First year	1.91	1.39	1.61	1.39			
Second year	2.39	1.38	1.78	1.35			
**Adaptation**					20.61 <0.001 (0.163)	NS	NS
First year	3.96	1.22	3.72	1.17			
Second year	4.36	1.10	3.95	1.17			

Note: F is the statistic representing the ratio of the mean square between groups to the mean square within groups (with 1 and 106 degrees of freedom unless stated otherwise); *p* is the *p*-value, and ${\eta_{p}}^{2}$ is the partial-eta-squared value, which indicates the effect size. NS = non-significant at a 95% confidence level.

**Table 6:** Repeated measures ANOVA results by year and age.

Scale	4–11 Years (n = 58)		12+ Years (n = 50)		$FF_{\left(1,106\right)}\;\;p\;({\eta_{p}}^{2})$		
	Mean	SD	Mean	SD	Time	Age	Time by Age Interaction
**Total scale**					40.88 <0.001 (0.278)	NS	NS
First year	2.76	1.00	2.52	1.17			
Second year	3.35	0.94	2.95	1.29			
**Basic skills**					35.20 <0.001 (0.249)	NS	NS
First year	1.73	1.58	1.28	1.52			
Second year	2.62	1.60	2.26	1.62			
**Advanced skills**					5.60 0.02 (0.05)	NS	F_(2,105)_ = 5.84 0.02 (0.052)
First year	1.49	1.23	2.09	1.50			
Second year	2.11	1.38	2.09	1.42			
**Adaptation**					20.54 0.008 (0.162)	7.34 0.001 (0.065)	NS
First year	4.09	1.04	3.57	1.31			
Second year	4.45	0.77	3.84	1.40			

Note: F is the statistic representing the ratio of the mean square between groups to the mean square within groups (with 1 and 106 degrees of freedom unless stated otherwise); *p* is the *p*-value, and ${\eta_{p}}^{2}$ is the partial-eta-squared value, which indicates the effect size. NS = non-significant at a 95% confidence level.

**Table 7:** Correlations between first- and second-year mean scores (n = 108).

Measure	r
AIM-2 total scale	0.730
Adaptation	0.813
Basic skills	0.476
Advanced skills	0.503

All the correlations are significant at *p* < 0.001.

## Data Availability

Data are contained within the article.

## References

[1] Becker B.E., Cole A.J. (2004). Comprehensive Aquatic Therapy.

[2] Marinho-Buzelli A.R., Bonnyman A.M., Verrier M.C. (2015). The effects of aquatic therapy on mobility of individuals with neurological diseases: A systematic review. Clin. Rehabil..

[3] Broach E., Datillo R. (1996). Aquatic therapy: A viable therapeutic recreation intervention. Ther. Recreat. J..

[4] Jorgić B., Dimitrijević L., Lambeck J., Aleksandrović M., Okičić T., Madić D. (2012). Effects of aquatic programs in children and adolescents with cerebral palsy: A systematic review. Sport Sci..

[5] Gehlsen G.M., Grigby S.A., Winant D.M. (1984). Effects of an aquatic fitness program on the muscular strength and endurance of patients with multiple sclerosis. Physiotherapy.

[6] Haung S., Viega R., Sila U., Reed E., Hines S. (1989). The effect of swimming in asthmatic children: Participants in a swimming program in Baltimore. J. Asthma.

[7] Hutzler Y., Chacham A., Bergman U., Szeinberg A. (1998). Effects of a movement and swimming program on vital capacity and water orientation skills of children with cerebral palsy. Dev. Med. Child Neurol..

[8] Harris S.R. (1978). Neurodevelopment treatment approach for teaching swimming to cerebral palsied children. Phys. Ther..

[9] Akinola B.I., Gbiri C.A., Odebiyi D.O. (2019). Effect of a 10-Week Aquatic Exercise Training Program on Gross Motor Function in Children with Spastic Cerebral Palsy. Glob. Pediatr. Health.

[10] Badawy W.M., Ibrahem M.B. (2016). Comparing the effects of aquatic and land-based exercises on balance and walking in spastic diplegic cerebral palsy children. Med. J. Cairo Univ..

[11] Roostaei M., Baharlouei H., Azadi H., Fragala-Pinkham M.A. (2017). Effects of Aquatic Intervention on Gross Motor Skills in Children with Cerebral Palsy: A Systematic Review. Phys. Occup. Ther. Pediatr..

[12] Hutzler Y., Bergman U. (2011). Facilitators and barriers to participation while pursuing an athletic career. Ther. Recreat. J..

[13] Pack S., Kelly S., Arvinen-Barrow M. (2017). “{I} think {I} became a swimmer rather than just someone with a disability swimming up and down”: {Paralympic} athletes’ perceptions of self and identity development. Disabil. Rehabil..

[14] Kārkliņa B., Declerck M., Daly D.J. (2013). Quantification of aquatic interventions in children with disabilities: A systematic literature review. Int. J. Aquat. Res. Educ..

[15] Canadian Red Cross Society (1983). Water Safety Instructor Guide and Reference: Water Safety Skills and Strokes for Safe Enjoyable Aquatics.

[16] Canadian Red Cross Society (1996). Canadian Red Cross Water Safety Services: Water Safety Instructor Manual.

[17] Gladish K. (2002). Swimming programs for infants and toddlers. Pediatrics.

[18] Thomas D.G. (2005). Swimming: Steps to Success.

[19] Killian K.J., Joyce-Petrovich R.A., Menna L., Arena S.A. (1984). Measuring water orientation and beginner swim skills of autistic individuals. Adapt. Phys. Act. Q..

[20] Killian K.J., Arena-Ronde S., Bruno L. (1987). Refinement of two instruments that assess water orientation in atypical swimmers. Adapt. Phys. Act. Q..

[21] Gelinas J.E., Reid G. (2000). The developmental validity of traditional learn-to-swim progressions for children with physical disabilities. Adapt. Phys. Act. Q..

[22] Jham B.C., Cannella D., Adibi S., Austin K., Allareddy V., Petrie C.S. (2018). Should pass/fail grading be used instead of traditional letter grades in dental education? two viewpoints: Viewpoint 1: Pass/fail grading improves learning experiences for students and viewpoint 2: Traditional letter grading provides objective evaluation for dental education. J. Dent. Educ..

[23] Ba-Ali S., Jemec G.B.E., Sander B., Toft P.B., Homøe P., Lund-Andersen H. (2017). The effect of two grading systems on the performance of medical students during oral examinations. Dan. Med. J..

[24] Yang L., Nong Q.-Q., Li C.-L., Feng Q.-M., Lo S.K. (2007). Risk factors for childhood drowning in rural regions of a developing country: A case-control study. Inj. Prev..

[25] Irwin C., Pharr J., Layne T., Irwin R. (2019). An investigation of youth swimming skills and method of instruction. Int. J. Aquat. Res. Educ..

[26] Conatser P., James E., Karabulut U. (2018). Adapted Aquatics for Children with Severe Motor Impairments. Int. J. Aquat. Res. Educ..

[27] Forde A., Zeman E., Clarke L. (2020). Effectiveness of an Intensive Drowning Prevention Program and Skills Retention by Children with and without Disabilities. Int. J. Aquat. Res. Educ..

[28] Murphy K.L., Hennebach K.R. (2020). A systematic review of swimming programs for individuals with autism spectrum disorders. J. Disabil. Stud..

[29] Di Paola P. (2019). The assessment of swimming and survival skills: Is your programme fit for its purpose?. Int. J. Aquat. Res. Educ..

[30] Vogt T., Staub I. (2020). Assessment of basic aquatic skills in children: Inter-rater reliability of coaches, teachers, students, and parents. J. Phys. Educ. Sport.

[31] Button C., Button A., Jackson A.-M., Cotter J., Maraj B. (2020). Teaching foundational aquatic skills to children in open water environments. Int. J. Aquat. Res. Educ..

[32] Santos-Garcia D.J., Rocca O., Navandar A., Murcia J.A.M. (2022). Measurement of aquatic competence in toddlers, infants, and children between 6 months and 14 years: A systematic review. Motricidade.

[33] Chan D.K.C., Lee A.S.Y., Macfarlane D.J., Hagger M.S., Hamilton K. (2020). Validation of the swimming competence questionnaire for children. J. Sports Sci..

[34] Sundan J., Haga M., Lorås H. (2024). Swimming competence of 9–10-year-old Norwegian primary school children: A cross-sectional study of physical education. Eur. Phys. Educ. Rev..

[35] Gorter J.W., Currie S.J. (2011). Aquatic exercise programs for children and adolescents with cerebral palsy: What do we know and where do we go?. Int. J. Pediatr..

[36] Chacham A., Hutzler Y. (2002). Water orientation scale for children and adolescents with disability: Validity and reliability. Bitnuaa.

[37] Getz M., Hutzler Y., Vermeer A. (2006). The Relationship between Aquatic Independence and Gross Motor Function in Children with Neuro-Motor Impairments. Adapt. Phys. Act. Q..

[38] Bar-Or O., Inbar O., Spira R. (1976). Physiological effects of a sports rehabilitation program on cerebral palsied and post-poliomyelitic patients. Med. Sci. Sports.

[39] American Educational Research Association, American Psychological Association, National Council on Measurement in Education (2014). Standards for Educational and Psychological Testing.

[40] Cronbach L.J., Meehl P.E. (1955). Construct validity in psychological tests. Psychol. Bull..

[41] Hutzler Y., Tesler R., Gilad A., Ng K., Barak S. (2023). 2022 para report card on physical activity of israeli children and adolescents with disabilities. Adapt. Phys. Act. Q..

[42] Jorgic B., Dimitrijevic L., Aleksandrovic M., Okicic T., Madic D., Radovanovic D. (2012). The swimming program effects on the gross motor function, mental adjustment to the aquatic environment, and swimming skills in children with cerebral palsy: A pilot study. Spec. Eduk. Rehabil..

[43] Ferreira C., Cunha M., Marta-Simões J., Duarte C., Matos M., Pinto-Gouveia J. (2018). Development of a measure for the assessment of peer-related positive emotional memories. Psychol. Psychother..

[44] Jacob M.E., Lo-Ciganic W.-H., Simkin-Silverman L.R., Albert S.M., Newman A.B., Terhorst L., Bilt J.V., Zgibor J.C., Schlenk E.A. (2016). The preventive services use self-efficacy (PRESS) scale in older women: Development and psychometric properties. BMC Health Serv. Res..

[45] Hatcher L., O’Rourke N. (2013). A Step-By-Step Approach to Using SAS for Factor Analysis and Structural Equation Modeling.

[46] Nunnally J.C., Bernstein I.H. (1994). Psychometric Theory.

[47] Schober P., Boer C., Schwarte L.A. (2018). Correlation Coefficients. Anesth. Analg..

[48] Sršen K.G., Vidmar G., Pikl M., Vrecar I., Burja C., Krušec K. (2012). Content validity and inter-rater reliability of the Halliwick-concept-based instrument “Swimming with Independent Measure”. Int. J. Rehabil. Res..

[49] Olaisen R.H., Flocke S., Love T. (2018). Learning to swim: Role of gender, age and practice in Latino children, ages 3–14. Inj. Prev..

[50] Fragala-Pinkham M., O’Neil M.E., Haley S.M. (2010). Summative evaluation of a pilot aquatic exercise program for children with disabilities. Disabil. Health J..

[51] Freudenheim A.M., Basso L. (2005). Organização temporal da braçada do nado crawl: Iniciantes “versus” avançados. Rev. Bras. Ciência Mov..

[52] Guo W., Soh K.G., Zakaria N.S., Hidayat Baharuldin M.T., Gao Y. (2022). Effect of resistance training methods and intensity on the adolescent swimmer’s performance: A systematic review. Front. Public Health.

[53] Ku B. (2020). The Effects of Motor Skill Interventions on Motor Skills in Children with Developmental Disabilities: A Literature Review. Asian J. Kinesiol..

[54] Gofer-Levi M., Silberg T., Brezner A., Vakil E. (2013). Deficit in implicit motor sequence learning among children and adolescents with spastic cerebral palsy. Res. Dev. Disabil..

[55] Donaldson M., Blanksby B., Heard N. (2010). Progress in precursor skills and front crawl swimming in children with and without developmental coordination disorder. Int. J. Aquat. Res. Educ..

[56] Dutia I., Curran D., Donohoe A., Beckman E., Tweedy S.M. (2022). Time cost associated with sports participation for athletes with high support needs: A time-motion analysis of tasks required for para swimming. BMJ Open Sport Exerc. Med..

[57] Petrea R.G., Moraru C.E., Rusu O.M., Rus C.M., Făgăraş P.S., Cristea F. (2023). Psycho-motor skills in swimming among children: Gender differences. Broad Res. Artif. Intell. Neurosci. (BRAIN).

[58] Syahrastani S. (2022). Differences in the three swimming style learning outcomes from gender. JPPI (J. Penelit. Pendidik. Indones.).

[59] Pitchford E.A., Leung W. (2021). Lower musculoskeletal fitness among children with disabilities, ages 6 to 15 years: 2012 NYFS: 657. Med. Sci. Sports Exerc..

[60] Wizer R.T., de Souza Castro F.A. (2021). Effects of age and experience on the development of aquatic competence in children aged 36 to 72 months. Motricidade.

[61] Rusdi, Lauh W.D.A. (2024). Swimming instructor confessions: Age and gender are most fastest in capturing swimming learning. Arus J. Sos. Hum..

[62] Watkins M.W. (2018). Exploratory factor analysis: A guide to best practice. J. Black Psychol..

[63] Hammer G.P., du Prel J.-B., Blettner M. (2009). Avoiding Bias in Observational Studies. Dtsch. Aerzteblatt Int..

[64] Powell J.T., Sweeting M.J. (2015). Retrospective Studies. Eur. J. Vasc. Endovasc. Surg..

[65] Rosenthal R. (1976). Experimenter Effects in Behavioral Research.

[66] Babad E.Y. (1985). Some correlates of teachers’ expectancy bias. Am. Educ. Res. J..

[67] Seifert L., Smeeton N.J., Dekerle J. (2020). A nonlinear pedagogy approach to promoting skill acquisition in young swimmers. High Performance Youth Swimming.

